# Where Do We Stand in Stem Cell Therapy for the Management of Diabetes Mellitus?—A Scientometric Research Trend Analysis from 1990 to 2020

**DOI:** 10.3390/bioengineering8110159

**Published:** 2021-10-26

**Authors:** Sathish Muthu, Madhan Jeyaraman, Naveen Jeyaraman, Ramya Lakshmi Rajendran, Prakash Gangadaran

**Affiliations:** 1 Department of Orthopaedics, Government Medical College and Hospital, Dindigul 624001, Tamil Nadu, India; drsathishmuthu@gmail.com; 2 Department of Biotechnology, School of Engineering and Technology, Sharda University, Greater Noida 201310, Uttar Pradesh, India; 3 Indian Stem Cell Study Group (ISCSG) Association, Lucknow 226010, Uttar Pradesh, India; naveenjeyaraman@yahoo.com; 4 Department of Orthopedics, School of Medical Sciences and Research, Sharda University, Greater Noida 201310, Uttar Pradesh, India; 5 Department of Nuclear Medicine, School of Medicine, Kyungpook National University, Daegu 41944, Korea; ramyag@knu.ac.kr; 6 BK21 FOUR KNU Convergence Educational Program of Biomedical Sciences for Creative Future Talents, Department of Biomedical Sciences, School of Medicine, Kyungpook National University, Daegu 41944, Korea

**Keywords:** stem cell, therapy, diabetes mellitus, scientometric research, trend analysis

## Abstract

Stem cell therapy has been considered a promising strategy in the management of both type I and type II diabetes mellitus (DM) because of its immunomodulatory and regenerative capability to restore the beta cell number and function. Various modalities of cellular therapy like transplantation of pancreatic islet cells, transplantation of pancreatic ductal stem cells, and mesenchymal stromal cell transplantation have been tried, and the modality is undergoing rapid advancements that may become the reality in the near future. In the course of its evolution, it is essential to have a comprehensive summary of the progress for a greater capacity to refine our future directives. With technological developments like data mining, graphic drawing, and information analytics combined with computational statistics, visualization of scientific metrology has become a reality. With a newer perspective, we intend to use scientometric tools including text mining, co-word analysis, word frequency analysis, co-citation analysis, cluster network analysis, to perform a systematic and comprehensive analysis of the research trend in stem cell therapy in the management of DM over the past three decades (1990–2020) and to identify the future research hotspots.

## 1. Introduction

The global prevalence of diabetes mellitus (DM) among adults over 18 years doubled to 8.5% from 4.7% in 1980 with a 5% increase in premature mortality, making it a major health problem worldwide [1]. Although the traditional therapy methods are aimed at maintaining the serum glucose levels through either exogenously administered insulin or oral anti-hyperglycemic medications, they often fail to achieve a balance in the glucose metabolism and result in hyperglycemic episodes responsible for fatal complications [2]. Stem cell therapy has been considered a promising strategy based on its immune-modulatory and regenerative capability in the management of both type I and type II DM by combating the auto-immune response affecting their production and the receptor insensitivity undermining their effect at the site of action [3,4].

Various modalities of stem cell therapy such as transplantation of pancreatic islet cells, transplantation of pancreatic ductal stem cells, and mesenchymal stromal cell transplantation from sources such as bone marrow or peripheral blood containing hematopoietic stem cells have been tried [5,6]. Various clinical trials have been conducted to analyze the efficacy and safety of these cellular therapy methods for their global practical applicability [7,8,9]. Although there were a few meta-analyses to support the use of stem cell therapy in type II DM [10,11,12], there have been a few that refuted their use in type I DM [13]. Hence, stem cell therapy for DM is evolving, and in the course of its evolution, it is essential to have a comprehensive summary of the progress to have a greater capability to refine our future directives. With technological developments like data mining, graphic drawing, and information analytics combined with computational statistics, visualization of scientific metrology has become a reality.

Scientometrics is a quantitative method of analyzing such an evolutionary process through various parameters like citation metrics, keyword, and author networks [14]. Scientometrics can visualize this panorama of information through knowledge maps to explore hotspots in research [15]. It has been widely used in other fields such as public health [16,17], artificial intelligence [18], and education research.

With a newer perspective, we intend to use scientometric tools including text mining, co-word analysis, word frequency analysis, co-citation analysis, and cluster network analysis to perform a systematic and comprehensive analysis of the research trend in stem cell therapy in the management of DM over the past three decades (1990–2020) and to identify the future research hotspots.

## 2. Methodology

### 2.1. Data Source

**Figure 1:**
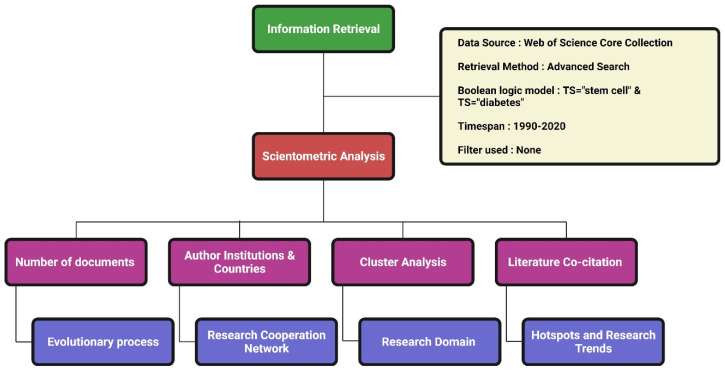
Scientometric analysis framework. Created with Biorender.com.

We used the Web of Science (WoS) as the source for data retrieval. Among the WoS databases, we used the WoS Core Collection with the indexes SCI-EXPANDED, SSCI, A&HCI, CPCI-S, CPCI-SSH, BKCI-S, BKCI-SSH, ESCI, CCR-EXPANDED, IC for data extraction. The detailed data retrieval strategy is given in Figure 1. Preliminary data were standardized with deduplication and merge functions in CiteSpace. The literature search date was 1 February 2021.

### 2.2. Data Visualization and Analysis

We used CiteSpace (5.7.R4) for scientometric and visualization analysis [19]. CiteSpace was used to visualize the structure, regularity, and distribution of research domains in stem cell therapy for DM and to analyze the article co-citation data to mine the knowledge clustering and citation space distribution. We also analyzed the co-occurrence among the additional research units such as cooperation among various authors, institutions, and countries in the field of stem cell therapy for DM. By consolidating the results of the analysis we built a comprehensive knowledge map elaborating on the emerging research trend with potential research domains in stem cell therapy for DM [20].

The scientometric analysis results are depicted as knowledge maps with the key parameters detailed as follows. The size of the nodes in the knowledge graphs indicates the frequency of authors, institutions, and countries, while the connection between them indicates that they are from the same article [21]. When two or more authors or institutions or countries were noted in the same article, it was considered a scientific cooperative relationship between the group of authors or institutions or countries [22].

The scientometric analysis uses certain parameters for evaluation. Silhouette is a parameter used to analyze the clustering effect in terms of the homogeneity of the network [23]. A value closer to 1 means higher homogeneity and a silhouette value of more than 0.7 shows high reliability. Burst is a measure of the frequency of citations acquired by an article in a period that indicates the impact and influence of the article on the subject based on the burst value and duration of burst, respectively [19]. Cluster analyses of the results were made using the log-likelihood ratio (LLR) text-mining algorithm in CiteSpace, and the research with common domains of metadata was clustered by the algorithm and presented as clustered knowledge maps, which were analyzed further for their characteristics compared to each other.

## 3. Results

**Figure 2:**
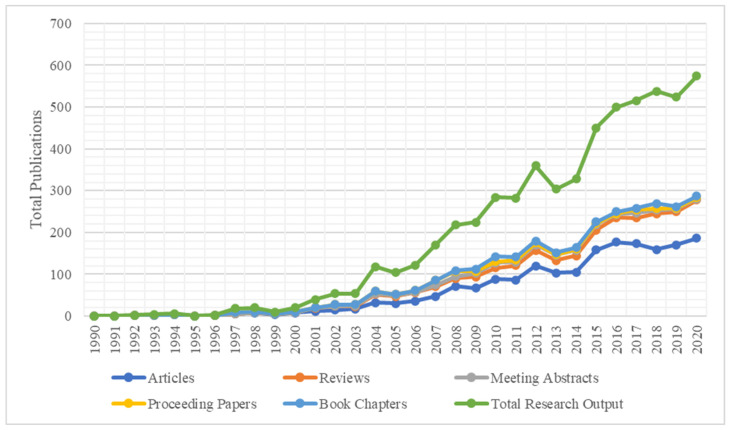
Scientific output in stem cell therapy for diabetes mellitus from 1990–2019.

We recovered 2901 published studies related to stem cell therapy for DM from the WoS Core Collection database, which included 1891 research articles, 752 reviews, 133 meeting abstracts, 88 proceeding papers, and 72 book chapters. Figure 2 shows the research output published every year on stem cell therapy for DM. The total publication count of research articles rose from an average of 3 per year in the first decade (1991–2010) to 144 per year in the last decade (2011–2020). There was an overall rising trend in the scientific production analyzing stem cell therapy for DM, as shown in Figure 2. An increased number of articles including clinical trials published on the use of stem cells for DM indicated increasing attention paid by the researchers in this field to improving the existing standard of care. It was also noted that there was a proportionate growing trend in all other types of research documentation such as original articles, reviews and proceedings papers, and meeting abstracts.

### 3.1. Journal Analysis

**Figure 3:**
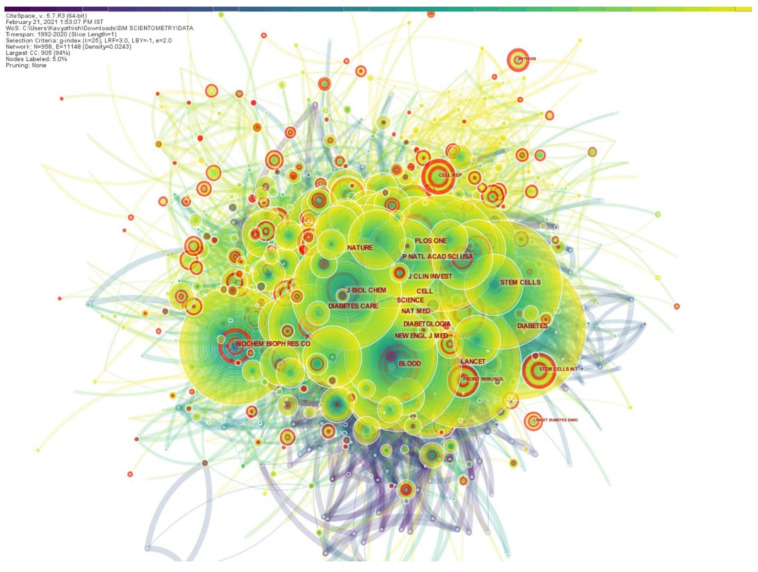
Journal citation network of stem cell therapy research for diabetes mellitus.

More than the number of articles the journal publishes, the number of literature citations of the published article can better reflect the importance and influence of a journal in the field. Hence, we used CiteSpace to analyze the list of journals where the retrieved results were published and generated a map of journals that cited them, as depicted in Figure 3. The journal citation network had 958 nodes and 11,148 links among them. The top five journals that published highly referenced articles on the use of stem cells for DM were *Diabetes, Proceedings of the National Academy of Sciences, Nature, Science* and *PLoS ONE*. As shown in Figure 3, there were a few journals with pink highlighting circles, such as *Frontiers in Immunology, Stem Cells International, Lancet Diabetes & Endocrinology*, around their nodes indicating that they had high burst values.

### 3.2. Scientific Cooperation Network Analysis

On author co-occurrence network mapping we found a network density of 0.0041, which denotes poor networking among the authors involved in stem cell research for diabetes. Bagher Larijani, Gian Paolo Fadini, and Kenneth Maiese were identified as the notable top researchers on the subject. We analyzed the co-institution network and found that researchers from institutes like Harvard University, University of Sao Paulo, and Stanford University made significant contributions to this field under analysis. On a similar note, countries like the USA, the Peoples Republic of China, and Italy were the top contributing countries toward research on this subject. However, the network among the institutions and countries involved in research on stem cell therapy for DM was poor with a low network density of 0.0024 and 0.0037, respectively, noted among them.

### 3.3. Co-Citation Analysis

Mutual citations of scholarly works govern the objective law of scientific development [24]. The top 10 key research articles that laid the foundation for using stem cells for DM management based on their citation frequency as burst values are presented in Table 1. It shows that the article by F.W. Pagliuca et al. [25], in which the properties of stem cell-derived beta cells such as insulin secretion upon glucose stimulation along with their resemblance to human beta cells by gene expression and ultrastructure was documented. It established the platform for the therapeutic utility of these properties toward DM management.

Moreover, the research by A. Rezania et al. [26] established that the insulin-secreting cells derived from human embryonic stem cells could be an effective alternative to the pancreatic progenitors or cadaveric islets that have been considered for the treatment of DM. It is also noted from Table 1 that these two articles had the burst timeline of 2015–2020. We also noted that research on similar grounds of stem cell-derived beta cells by H.A. Russ et al. [27], J.R. Millman et al. [28], and A.J. Vegas et al. [29] were included in the top 10 articles with a high citation frequency burst from 2017–2020, making it a research hotspot and a potential research frontier of future.

The utility of ductal cells and pancreatic cells as sources of cellular therapy was the research focus of the last decade. It was evident from the burst range of articles by S. Bonner-Weir et al. [5], H. Zulewski et al. [30], and V.K. Ramiya et al. [31], which dealt with using expanded ductal cells and pancreatic islet cells as a source of cellular therapy, had a citation burst range from 2001–2008.

Rajagopal et al. [32] described the limitations of various methods of estimation of islet cell differentiation of the embryonic stem cells, while Millman et al. [28] reported a scalable in vitro production of functional stem cell-derived beta cells from type 1 diabetes patients. Vegas et al. [29] reported long-term glycemic control in polymer-encapsulated human stem cell-derived glucose-responsive insulin-producing beta cells in an animal model.

**Table 1:** Top 10 articles on stem cell therapy for diabetes based on the strength of their citation frequency burst.

Sl.No.	Key Publications	Year	Strength	Begin	End	1990–2020
1	Pagliuca FW et al. [25]	2014	62.5	2015	2020	▂▂▂▂▂▂▂▂▂▂▂▂▂▂▂▂▂▂▂▂▂▂ ▂ ▃▃▃▃▃▃
2	Rezania A et al. [26]	2014	49.73	2015	2020	▂▂▂▂▂▂▂▂▂▂▂▂▂▂▂▂▂▂▂▂▂▂ ▂ ▃▃▃▃▃▃
3	Bonner-Weir S et al. [5]	2000	31.55	2001	2008	▂▂▂▂▂▂▂▂ ▂ ▃▃▃▃▃▃▃▃ ▂▂▂▂▂▂▂▂▂▂▂▂
4	Zulewski H et al. [30]	2001	30.16	2002	2008	▂▂▂▂▂▂▂▂▂ ▂ ▃▃▃▃▃▃▃ ▂▂▂▂▂▂▂▂▂▂▂▂
5	Ramiya VK et al. [31]	2000	27.86	2001	2008	▂▂▂▂▂▂▂▂ ▂ ▃▃▃▃▃▃▃▃ ▂▂▂▂▂▂▂▂▂▂▂▂
6	Russ HA et al. [27]	2015	27.53	2016	2020	▂▂▂▂▂▂▂▂▂▂▂▂▂▂▂▂▂▂▂▂▂▂▂ ▂ ▃▃▃▃▃
7	Rajagopal J et al. [32]	2003	25	2004	2009	▂▂▂▂▂▂▂▂▂▂▂ ▂ ▃▃▃▃▃▃ ▂▂▂▂▂▂▂▂▂▂▂
8	Millman JR et al. [28]	2016	22.88	2017	2020	▂▂▂▂▂▂▂▂▂▂▂▂▂▂▂▂▂▂▂▂▂▂▂▂ ▂ ▃▃▃▃
9	Assady S et al. [33]	2001	22.83	2002	2008	▂▂▂▂▂▂▂▂▂ ▂ ▃▃▃▃▃▃▃ ▂▂▂▂▂▂▂▂▂▂▂▂
10	Vegas AJ et al. [29]	2016	22.56	2016	2020	▂▂▂▂▂▂▂▂▂▂▂▂▂▂▂▂▂▂▂▂▂▂▂▂ ▃▃▃▃▃

▃▃: burst period; ▂▂: article timeline; ▂▂: citation period.

### 3.4. Cluster Analysis

**Figure 4:**
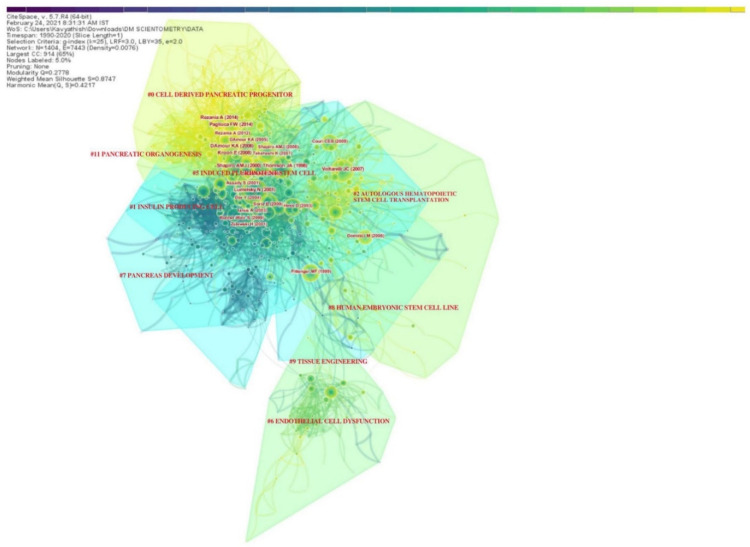
Article co-citation network highlighting the various research clusters on the use of stem cells for management of diabetes mellitus and their research interactions.

**Figure 5:**
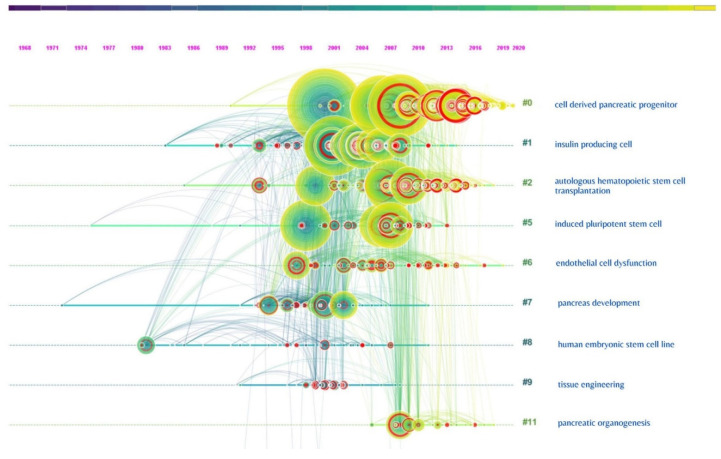
Timeline summary of the various clusters and their interactions on the use of stem cells for management of diabetes mellitus.

The various research domains on utilizing stem cells in the management of DM were categorized under various research clusters based on the total number of similar articles included in them, as shown in Table 2. There were 9 clusters noted in the network, with the largest cluster having 210 articles in it and smallest with 27 articles. Almost all the clusters had a high similarity index depicted by the silhouette values. The labels used in the clusters were from the LLR data mining algorithm in CiteSpace. The interactions between various research clusters are shown in Figure 4 and Figure 5, and the top 5 clusters that form the research base are discussed below:

### 3.5. Cluster #0 Cell-Derived Pancreatic Progenitor

This was the largest cluster with 210 linked research articles. The mean period of the articles in the cluster was 2013. The top-cited articles of this cluster included articles by A.M.J. Shapiro et al. [34], E. Kroon et al. [35], K.A. D’Amour et al. [36], F.W. Pagliuca et al. [25], and A. Rezania et al. [26]. These articles established the platform for utilizing cell therapy in the management of DM. This cluster mainly dealt with the potential of stem cell-derived beta cells and the key mechanisms to modulate their function to obtain an optimal cell base for practical utility in the management of DM. The cell sources described in this cluster included pluripotent human embryonic stem cells and mesenchymal stem cells.

### 3.6. Cluster #1 Insulin-Producing Cell

This was the second largest cluster with 194 linked research articles. The mean period of the articles in the cluster was 2002. The top-cited articles in the cluster included articles by N. Lumelsky et al. [37], Y. Dor et al. [38], S. Bonner-Weir et al. [5], and Soria et al. [39]. This cluster of articles probed the alternate sources of insulin-producing cells other than stem cells, such as pancreatic ductal cells and islet cells. They also explored the differentiation dynamics of adult pre-existing beta cells.

### 3.7. Cluster #2 Autologous Hematopoietic Stem Cell Transplantation

This was the third largest cluster with 153 linked research articles. The mean period of the articles in the cluster was 2008. The top-cited articles in the cluster included articles by J.C. Voltarelli et al. [40], M. Dominici et al. [41], C.E.B. Couri et al. [42], M.F. Pittenger et al. [43], and R.H. Lee et al. [44]. This cluster of articles probed the utility of hematopoietic stem cells as a source of progenitors for differentiation into insulin-secreting islet cells. They also analyzed the homing property of multipotent hematopoietic stem cells to repair the damage caused by the DM in pancreatic islets and renal glomerulus.

### 3.8. Cluster #5 Induced Pluripotent Stem Cell

This cluster was formed by 66 linked research articles with a mean period of 2005. Notable articles in the cluster included the contribution by J.A. Thomson et al. [45], K. Takahashi et al. [46,47], and J. Yu et al. [48]. This cluster dealt with the utilization of the reprograming a differentiated, somatic cell such as a fibroblast into a patient- and disease-specific pluripotent stem cell of choice in the management of DM. Induced pluripotent stem cells possess similar morphology, proliferative capacity, and genetic composition to that of the embryonic stem cell [44], and hence, they have been considered as a potential source of cell therapy in DM. The stage of such a pluripotent stem cell that is ideal in the management of DM needs further exploration.

### 3.9. Cluster #6 Endothelial Cell Dysfunction

This cluster was formed by 56 articles in the network with a mean publication period of 2007. The key research articles included the contribution of T. Asahara et al. [49], O.M. Tepper et al. [50], C.J.M. Loomans et al. [51], and G.P. Fadini et al. [52,53]. This cluster elucidated the various forms of pathogenesis in the impairment of the endothelial progenitor cell function in diabetes that manifests as peripheral vascular disease. Hence, it is imperative to address these issues with the potential of cellular therapy if we aim to manage the complications of the disease for chronic cases.

## 4. Discussion

Based on the research output analyzed, there was a rising trend nominally year by year in the published literature. We also noted a proportionate increase in the number of proceedings papers and meeting abstracts, which showed that there was an increase in the number of international academic activities on cellular therapy for the management of DM. This emphasized the increase in international attention to the innovation and improvisation of the existent standards of care. With the increase in the proportion of the aging population [54], there are rising concerns for advancement in the management of DM.

This article explored the research cooperation in cellular therapy for DM from three perspectives, namely, small—author cooperation network, intermediate—institutional cooperation network, and large—national cooperation network. We did not find a strong network among the researchers in this field, and this needs further strengthening to effectively utilize the human resources and reap the benefits of collaborative research.

## 5. Emerging Trends

Keywords reflect the core research content, research themes, and the main direction of research involved in the article [55]. With text mining and scientometric techniques such as keyword co-occurrence analysis, we can spot the trends in research and research hotspots in the field of interest [56]. We unveiled emerging trends by analyzing the keyword co-occurrences and literature co-citations on stem cell therapy for DM. From the result of this analysis, the major research domains involving stem cell therapy for DM currently involve identification of the ideal stem cell type from among varied sources such as hematopoietic stem cells, mesenchymal stem cells, induced pluripotent stem cells, or human embryonic stem cells for optimal effects at the site of action.

Despite the varied sources of stem cells, such as bone marrow, adipose tissue, and umbilical cord, identification of the ideal, safe, and effective source of stem cells is crucial. Although Carlsson et al. [57] did not report any difference in the exogenous insulin requirement or HbA1c or c-peptide level with bone marrow source, a significant difference was noted by Hu et al. [58] from the umbilical cord source stem cell. Moreover, adipose tissue that provides a high yield of mesenchymal stem cells has also been tried in this setting on animal models and found to be beneficial, which requires further exploration for clinical benefit [59].

The critical effect of the transplanted environment, such as hyperglycemia, hyperinsulinemia, and metabolic alterations of the native effects of stem cells, needs further exploration [60]. With selective sorting of the “youthful” MSCs that express positivity for stage-specific embryonic antigen-4 (SSEA-4), it is possible to establish personal stem cell banks that will allow serial infusions of “rejuvenated” MSCs for treating age-related diseases [61]. Apart from these methods, genetically engineered stem cells could provide enhanced therapeutic potential for diseases such as bone disease, cardiovascular diseases, and auto-immune diseases. However, there is a paucity of studies to evaluate their efficacy and safety for clinical use.

## 6. Future Prospects

Y. Zhang et al. [10] and G. Hwang et al. [13] in their meta-analyses of clinical trials noted a disparity in the results of stem cell therapies from different sources among type I and type II diabetes. They noted improved HbA1c levels and c-peptide levels in patients treated with bone marrow-derived hematopoietic stem cells (HSCs) compared to MSCs in patients of type I diabetes while type II diabetes patients responded well to MSC therapy [10,13]. Hence, future research to address the ideal source of stem cell therapy for the variants of DM is needed.

Research fronts that need further exploration before adapting stem cell therapy for practical utility involve optimization of stem cell-derived beta cell function for therapeutic effect by identifying the appropriate dosage, and route of administration for targeted action [62]. Special concern should be given to the effect of the native milieu in newly diagnosed and longstanding cases of DM on the transplanted stem cells. Further exploration into the homing and differentiation kinetics of the transplanted stem cells into insulin-secreting beta cells in vivo, along with the outcome measures like efficacy, safety, risks, and reliability of this modality, is needed to enhance the utilization of the therapy into clinical use. Apart from translating stem cell therapy for insulin secretion and metabolic homeostasis, utilization of the regenerative capacity of the stem cells to combat the endothelial dysfunction inherent to the disease would bring a holistic approach to disease management.

Despite the strengths, there are still some limitations to this study. From the perspective of research data used in the article, we used only the WoS Core Collection database. We did not take the gray literature such as non-published conference documents, scientific reports, dissertations, scientific archives, etc., into consideration as a data source. Hence, we warn readers to interpret the results of our analysis with caution considering the source of data utilized for the analysis. From a visual analysis perspective, we did not incorporate all the available information into the knowledge map.

## 7. Conclusions

We performed a comprehensive review of the available literature and analyzed the research trend over the past three decades in stem cell therapy for DM. Stem cell therapy has evolved into a promising strategy in the management of DM. Despite establishing its clinical efficacy and safety in some phase I/II trials, utilization of stem cell therapy for clinical use needs further research and standardization. Current research hotspots in stem cell therapy for DM involve identifying and optimizing the ideal source of stem cells for differentiation into insulin-secreting beta cells at the site of action. Apart from identifying the optimal source and understanding the stem cell function, future research into the appropriate dosage, route, and in vivo differentiation kinetics is also needed. Despite translating stem cell therapy for insulin secretion and metabolic homeostasis, utilization of the regenerative capacity of the stem cells to combat the endothelial dysfunction inherent to the disease would bring a holistic approach to disease management.

## Figures and Tables

**Table 2:** Cluster summary of co-citation network.

Cluster ID	Size	Silhouette	Mean (Year)	Label
0	210	0.83	2013	cell-derived pancreatic progenitor, mesenchymal stem cell
1	194	0.764	2002	insulin-producing cell, islet neogenesis
2	153	0.903	2008	autologous hematopoietic stem cell transplantation
5	66	0.894	2005	induced pluripotent stem cell
6	65	0.982	2007	endothelial cell dysfunction
7	56	0.913	1998	pancreas development, developmental biology
8	43	0.971	1997	human embryonic stem cell line
9	41	0.952	2000	tissue engineering
11	27	0.937	2012	pancreatic organogenesis, regeneration strategies

## Data Availability

Data will be shared upon request.
